# Building a relevant biomedical graduate program: from review to reform

**DOI:** 10.1186/s12909-026-09243-2

**Published:** 2026-04-22

**Authors:** Lisa Eunyoung Lee, Alaa Youssef, Sobiga Vyravanathan, Nicole Harnett

**Affiliations:** 1 Institute of Medical Science, University of Toronto, Toronto, ON Canada; 2 Centre for Artificial Intelligence in Medicine and Imaging, Stanford University, Stanford, CA USA; 3 Department of Radiation Oncology, University of Toronto, 610 University Avenue, Toronto, ON M5G 2C4 Canada

**Keywords:** Curriculum development, Program evaluation, Medical science, Biomedical education, Graduate education

## Abstract

**Background:**

Program evaluation is critical for ensuring that graduate programs remain responsive, effective, and aligned with the evolving needs of students, faculty, and the broader scientific community. At a major Canadian institution, we aimed to conduct a comprehensive evaluation to assess how well our program meets stakeholder needs and to identify curricular gaps and actionable recommendations across our biomedical and clinical research graduate programs.

**Methods:**

A mixed-methods approach was used, guided by the U.S. Centers for Disease Control and Prevention (CDC) Program Evaluation Framework. Data were collected through surveys, interviews, and a focus group, which included a combination of students, alumni, supervisors, and/or faculty members. The evaluation assessed the curriculum structure, program strengths, and areas for improvements.

**Results:**

The findings, when considered together, demonstrated that the student learning experience is shaped by numerous factors beyond course content. Synthesis of the results produced 26 recommendations in three main categories of factors, including course content and experiences, infrastructure and support, and faculty and supervisor engagement. In addition, we identified factors that enabled successful program evaluation, including overt institutional support of the evaluation process including commitment to establishment of a recurring cycle of implementation that aligned with other existing quality assurance processes, and establishment of formal activities to convert findings to actionable initiatives and to monitor progress for each.

**Conclusion:**

This evaluation demonstrated how the CDC Program Evaluation Framework can be used to enable a systematic, data-driven process that translated stakeholder feedback into actionable recommendations to support meaningful program refinements in response to identified needs regarding program modernization designed to better meet new realities for graduate students. Ongoing evaluation will be essential to monitor the impact of implemented changes and ensure alignment between graduate biomedical education and evolving scientific and workforce needs.

**Supplementary Information:**

The online version contains supplementary material available at 10.1186/s12909-026-09243-2.

## Background

The rapid advancement of scientific knowledge presents a challenge to traditional biomedical research-based graduate education paradigms, which often struggle to keep pace with emerging discoveries and evolving needs. To ensure students develop the skills and expertise required for diverse career paths, graduate education must remain dynamic and responsive [1]. Research from our own institution [2] and published work from the past decade [3, 4] clearly demonstrate that the traditional academic career path is no longer the dominant choice for graduates, with many pursuing roles in industry, biotechnology, healthcare consulting, government, non-profit, and entrepreneurship. Reflecting Biggs’ theory on constructivist alignment [5], a static curriculum risks becoming outdated, leaving students underprepared to navigate the complexities of modern biomedical and clinical research.

This challenge has become increasingly evident at our institute – the largest graduate unit within the Faculty of Medicine at a major Canadian university. Established in 1968, the institute aspires to be a global leader in biomedical graduate education, advancing human health through translational research that bridges scientific discovery with clinical application [6, 7]. During the time period of this study, there were over 700 faculty members and 500 graduate students across 11 clinical departments. Between 2000 and 2020, the institute graduated 2,375 students. The institute has seen growing diversity among both faculty and students [6, 7], and as such, new scientific niches are rapidly emerging, that when coupled with the fact that graduates are seeking careers beyond the traditional academic sector [3], it is clear that traditional graduate education models need to be monitored in order to assure ongoing alignment with the needs of the students and the system into which they transition.

In 2018, as part of the university’s quality assurance program, an external review of the institute was conducted by a panel of experts from outside the university, who held equivalent positions in graduate programs at their own institutions. The reviewers’ final report offered objective recommendations addressing key challenges raised by students, particularly regarding limited access to relevant and up-to-date educational and training opportunities. This external review highlighted the need for a structured effort to assess how well the program was meeting the needs of its diverse stakeholders, which would be instrumental for informing future renewal activities. A project team, including the Curriculum Director (NH) and two volunteer graduate students, was commissioned by the institute Director to conduct an institute-wide program evaluation to further explore the recommendations of the external review panel.

In this paper, we describe a mixed-methods evaluation of how well the institute’s programs meet the needs of key stakeholders, including students, alumni, supervisors (faculty members who supervise student research), and faculty members (those involved in teaching and academic leadership). Guided by the U.S. Centers for Disease Control and Prevention (CDC) Program Evaluation Framework [8, 9], we present our findings and set of recommendations (CDC Step 5) along with the institute’s plan for implementation (CDC Step 6). Detailed reporting on progress on specific actions related to individual recommendations is beyond the scope of this paper; however, initial action plans will be highlighted.

### CDC program evaluation framework

**Fig. 1:**
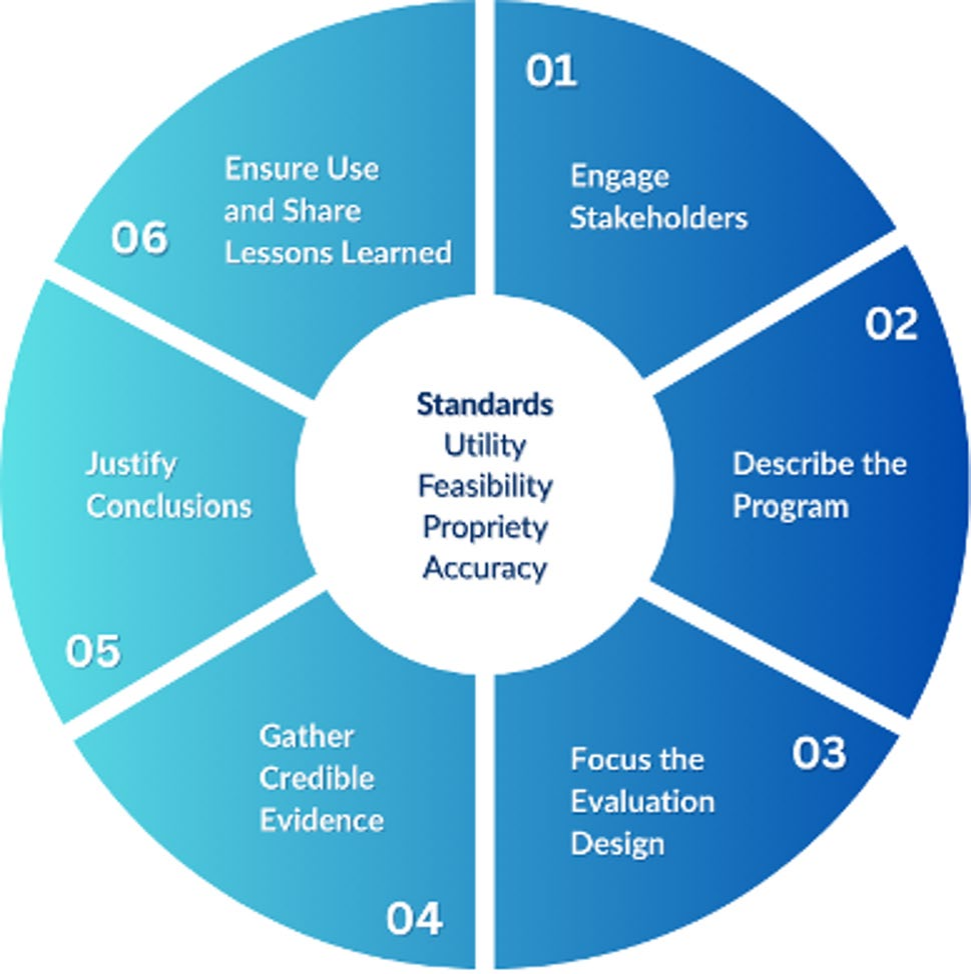
The CDC program evaluation framework

The CDC Program Evaluation Framework [8] is a systematic approach designed to assess and improve the effectiveness of a program (Fig. 1). It consists of six steps: (1) engage stakeholders, (2) describe the program, (3) focus the evaluation design, (4) gather credible evidence, (5) justify conclusions, and (6) ensure use and share lessons learned. While originally developed to guide public health professionals in program evaluation, this framework is widely applicable across various fields, including graduate education, and serves as a practical guide for understanding program impact and building evidence to inform data-driven improvements that ensure the program meets its goals. While developed in 1999, the framework was updated by Kidder et al. [10] in 2024 to reflect more contemporary understandings in the field of evaluation.

## Methods

### Audience and goals (step 1: engage stakeholders)

The internal program evaluation involved students, alumni, supervisors, and faculty members to ensure that diverse perspectives were incorporated throughout the process. The primary goal of the evaluation was to identify gaps and opportunities for improvement in structure, content, and overall student experience within the institute’s graduate research programs. This aimed to ensure that the curriculum remains responsive to evolving advances in science, and aligned with the needs of graduate students, while also confirming that appropriate supports are in place to optimize student learning.

### Document review (step 2: describe the program)

In order to define and contextualize the institute’s graduate programs, we reviewed institutional strategic priorities, university requirements, employer expectations, graduate employment data, prior stakeholder feedback, and relevant policies and procedures.

### Data collection (step 3: focus the evaluation design, step 4: gather credible evidence)

To gain a comprehensive understanding of the factors influencing the degree to which the program was meeting the needs of its stakeholders, a mixed-methods approach using an open voluntary response sampling method was used to conduct the program evaluation including surveys, interviews, and a focus group (Supplementary Files 1 to 5). Data collection took place between 2020 and 2021.

#### Surveys

The project team developed surveys using the Research Electronic Data Capture (REDCap) platform (https://project-redcap.org/), based on external review findings and informed by document review findings (more detailed information per group can be found below). Data were extracted by a single researcher (AY) using pre-defined fields and response options. Data quality was assessed through logic checks across related variables to identify inconsistencies, as well as review of outliers for plausibility. Identified discrepancies were reviewed and resolved by AY and independently checked by another researcher (graduate student member or NH) prior to analysis. The surveys were pilot tested by students and members of the institute’s leadership team to ensure face and content validity. Participation in each survey was voluntary and at one time point. All survey data were analyzed using IBM SPSS Statistics software (https://www.ibm.com/products/spss-statistics), and descriptive statistics were calculated to summarize survey responses.

The student survey assessed current students’ perspectives on their program requirements, course access, learning gaps, and experiences with the Collaborative Specialization Programs (CSPs) (interdisciplinary programs that enable students to gain specialized expertise alongside their main graduate studies). The survey was distributed through the institute’s online newsletter between July and August 2020. Students who provided their contact information received a $5 gift card from a local vendor.

The alumni survey focused on support for career planning and assessed the strengths and weaknesses of the CSPs. The survey was distributed through an online community platform for the Faculty of Medicine and through targeted emails from the Faculty of Medicine Advancement Office (March 2021). The faculty/supervisor survey explored the level of faculty engagement within the institute and their views on gaps in graduate research training. The survey was distributed through an email from the institute’s Director in July 2020.

#### Interviews

Interviews were conducted with faculty/supervisors, who indicated their willingness to participate and provided their contact information in the online survey. The project team developed the interview guide based on the survey results to gather more detailed insights on identified themes. The interview script was piloted on two internal faculty leaders to establish timing and content validity. Interviews were recorded and transcribed verbatim.

Thematic analysis was performed according to Braun and Clarke’s six-step approach [11], which comprises familiarization, coding, theme development, review, definition, and write-up. Analysis proceeded through:

1. familiarization with transcripts;
2. initial coding completed by one team member (AY) and reviewed by a second graduate student member;
3. development of candidate themes and subthemes;
4. refinement through team discussion and consensus building; disagreements adjudicated by the senior author (NH);
5. final theme definition and selection of illustrative quotations; and.
6. writing up the final findings and conclusions.

Coding was performed manually. AY performed the first analysis, which was reviewed by a graduate student member and NH. The project team discussed the analysis at consensus meetings and themes were revised iteratively until consensus was reached. When relevant, the interview guide questions were updated to reflect new findings.

#### Focus group

A focus group of students was held to gain further insights into student experiences, primarily related to their involvement in the CSPs. The invitation was distributed through the institute’s online newsletter and the Student Association. All students who expressed interest were invited to join the focus group. Similar to the interviews, the project team discussed any modifications to existing themes or the addition of new emerging themes. When relevant, the original focus group questions were updated to reflect new findings. The session was recorded, transcribed, and combined with the notes taken by researchers during the event. Participants were compensated with a $10 gift card from a local vendor. Thematic analysis followed the same Braun and Clarke six-step approach [11] that was used for the faculty/supervisor interviews.

#### Retreat

A one-day retreat was held in 2022 with participation from members from all relevant stakeholder groups with the goal of articulating a plan to address the final recommendations. It was the culminating step in this program evaluation.

## Results

### Participation

Surveys were completed by students (*n* = 128), alumni (*n* = 71), and faculty/supervisors (*n* = 83). Precise survey response rates cannot be accurately calculated because calls for volunteers were distributed through listservs and general communication channels such that recipient numbers are unknown. Estimated response rates are provided where possible. Eleven faculty/supervisors (13% of those who completed the survey) completed the one-on-one interview and seven students participated in the focus group.

### Findings by data source

#### Document review

The document review helped characterize the program’s structure and intended goals and expectations. Notably, it revealed a lack of clearly articulated program goals and objectives, and curricular gaps that did not fully align with students’ evolving needs or advances in biomedical science. These findings highlighted the need for a comprehensive evaluation to inform program renewal activities.

#### Student survey

A total of 128 students completed the curriculum needs assessment survey between July and August 2020 (Table 1). Of these, 84 (66%) were Master of Science (MSc) students and 44 (34%) were Doctor of Philosophy (PhD) students. Students’ research areas included clinical sciences, basic sciences, translational research, health systems and services, and population health. Responses were received from students in all nine of the institute’s research units. These respondent characteristics were distributed similarly to those of the overall student population.

**Table 1:** Characteristics of student participants (*n* = 128)

Graduate Program Type (*n*, %)		
Master of Science (MSc)		84 (66%)
Doctor of Philosophy (PhD)	PhD with a defended research-based MSc degree	28 (22%)
	PhD direct entry without a MSc degree	13 (10%)
	Doctor of Medicine (MD)/PhD	3 (2%)
Research Area (n, %)		
Clinical Sciences		56 (44%)
Basic Sciences		34 (27%)
Translational Research		26 (20%)
Health Systems and Services		2 (2%)
Population Health		3 (2%)
Other		7 (5%)

Of importance to the issue of availability of relevant course content in the institute, 26% of students completed all course requirements outside the institute. Reasons given included inability to access all or some course content relevant to their research area at the institute (44%), prompting them to seek courses in other departments in the university. When asked about specific unmet learning needs, 39% of students reported a need for professional development support, and 61% reported a need for courses that advanced their practical skills, particularly in statistics, coding, and grant writing.

#### Alumni Survey

A total of 71 former students completed the alumni survey. Respondents included 26 MSc (37%) and 45 PhD (63%) graduates. Alumni rated the extent to which their graduate training program prepared them for their current jobs as follows: strongly agree (23%), agree (58%), disagree (13%), and strongly disagree (7%). When asked which skills they wished to have developed further during their student experience, a number of skills were mentioned as summarized in Table 2, with the highest responses for scientific writing (54%) and statistical analysis (51%). In addition, 43 (61%) respondents perceived insufficient networking and career planning support at the institute.

**Table 2:** Skills alumni wished to develop further (option to select all that apply) (*n* = 71)

Skill	Response (*n*, %)
Scientific Writing (Thesis, Manuscript, Grant)	38 (54%)
Statistical Analysis	36 (51%)
Critical Thinking	35 (49%)
Teaching	31 (44%)
Communication	30 (42%)
Method Techniques	28 (39%)
Coding	27 (38%)
Conflict Resolution	23 (32%)
Other	4 (6%)

#### Faculty/supervisor survey

A total of 83 faculty/supervisors completed the supervisor survey. The degree of graduate student supervision experience reported by respondents is as follows: 56% of respondents have supervised 1–5 of our institute’s MSc students and 60% have supervised 1–5 of our institute’s PhD students. Further 20% have supervised 6–10 MSc students and 9% have supervised 6–10 PhD students.

When asked to suggest opportunities to improve curricular opportunities for students, faculty/supervisors identified the following areas as important for student development and competency: statistical and research methodological skills (80%), grant/manuscript writing (60%), writing/presentation skills, general/specific knowledge, and analytical skills (50%), and professional development and networking skills (26%). In general, faculty/supervisors rated students’ ethical conduct, teamwork, organization skills, and thesis progress very highly.

#### Faculty/supervisor interview

A total of 11 interviews were held with faculty/supervisors who volunteered through the online survey to participate. Participants included faculty from basic science (*n* = 4), translational research (*n* = 2), and clinical research (*n* = 5) disciplines from five of the 11 clinical departments: psychiatry (*n* = 4), cardiovascular sciences (*n* = 2), regenerative medicine (*n* = 1), obstetrics and gynecology (*n* = 1), surgery (*n* = 3). The length of institute membership ranged from 2 to 25 years and the range of years supervising graduate students (including MSc and PhD students) was 0 to 24 years.

In response to the question, “What are some curriculum gaps (academic or professional) that the institute needs to address? Why?”, the majority of participants spoke to core academic research skills from study design to manuscript preparation and to broader research related themes like ethics and artificial intelligence in research.

For example, a participant said: “*I think areas where students struggle is with statistics and writing manuscripts*,* grants*,* and thesis*,* and putting together presentations. A lot of that we bridge through lab mentorship*,* but more structured support would be of benefit to students.*”

Four main themes emerged from the thematic analysis of the interviews regarding opportunities to improve the institute’s programs, including creating clusters/streams to facilitate the ability for students and faculty to find their ‘identity’ within the large institute, finding ways to enhance faculty engagement through activities and communication strategies, improving the institute’s profile using communication with and presence in the relevant clinical department, and augmenting student support for accessing important discipline-specific content and professional development. This is highlighted in the quote from another participant: “*I think the [institute] does stay hard to stay engaged with students and faculty. It is difficult because the [institute] is a very large diverse graduate department. I would not say this is a weakness per se*,* but more of an evolving landscape to potentially re-examine how we might consider the engagement of faculty and students. The use of virtual technology as a hybrid meeting may be an opportunity to improve presence and engagement at the [institute].*”

#### Student focus group

Of the 13 students invited, seven students participated in a one-hour focus group focused primarily on the CSPs available to students in the institute. All students were currently enrolled in a CSP (4/7 Neuroscience, 3/7 Cardiovascular Sciences). Students stated the benefits of being in a CSP were that it was nice to have on their transcript to convey their area(s) of interest, that the requirements were not heavy, that there was a greater sense of community amongst students in the CSPs, and that in some cases students needed the CSP to gain access to content relevant to their research. The challenges that arose in the focus group included that students did not feel “specialized” at the completion of the CSP work, and that some content in the CSP was not relevant to specific students’ research focus as highlighted in the quote from a participant: “*[Having a CSP] sounds more prestigious than it really is. I feel that to have a ‘specialization*,*’ I should be learning beyond this and really pushing my knowledge in the field.*”

When asked for suggestions on how to improve CSPs, they suggested including students in any efforts to modify CSPs in the future and to consider making acceptance into CSPs competitive and consider assigning awards or grants to the acceptance.

### Summary analysis and key findings (step 5: justify conclusions)

After completing the evaluation of data from 300 responses from various stakeholders, the project team formulated draft recommendations based on the findings and presented these to the institute’s Leadership Committee and the Curriculum Committee in December 2021. The discussions led to 26 finalized recommendations reflecting the contemporary needs of the institute’s diverse stakeholder groups, organized under three primary themes:

1. Content and Experiences (14 recommendations): This area of focus relates to what students can access to develop their knowledge, skills, and judgment. Participants in this study suggested several strategies to improve the relevance of the institute’s programming. These suggestions represent not only optimizing course content and addressing emerging fields, but also opportunities to gain additional competencies during their graduate degree through intra and extra-curricular activities.
2. Infrastructure and Support (6 recommendations): Beyond curriculum, the systems, processes, and people in place to support students are critical to a successful and meaningful educational experience. This infrastructure shapes the learning environment and culture that the students are embedded in. Students, alumni, and faculty/supervisors identified many ways that support systems can be imagined and improved.
3. Faculty/Supervisor Engagement and Support (6 recommendations): The institute has grown immensely since inception and as such includes a rich and diverse pool of faculty and supervisors who play vital roles in the educational and professional development and experience of our students. Faculty and supervisors’ engagement in the institute must be maximized to ensure optimal programming and experiences.

### Step 6 (ensure use and share lessons learned)

While a full report on the utilization of the recommendations is beyond the scope of this paper, it can be reported that the institute took its first step towards action by convening a one-day retreat in January 2022 involving representation from all stakeholder groups, including institute leadership, curriculum leads, staff, students, faculty and supervisors, and alumni (*n* = 22). They convened to discuss each recommendation and develop actionable plans to address the recommendations. Recommendations were assigned to owners including ideas for potential solutions and expected timelines (short (0–3 months), mid (3–6 months), long-term (more than 6 months). Nine recommendations were identified as short-term goals. Ten recommendations were determined to be mid-range goals. Seven recommendations were deemed to be long-term goals. A shared document was created to track progress and identify challenges that needed addressing by the institute leadership.

## Discussion

The findings from the program evaluation were highly informative and catalyzed several initiatives to improve the overall student learning experience in our institute. While the primary objective of the evaluation was to inform curricular renewal, the results highlighted that the student learning experience is shaped by factors extending beyond course content alone.

Past studies have identified multiple contributors to curriculum quality, including program and dissertation length, the extent of supervisory support, student involvement in quality assurance, and students’ perceptions of teaching effectiveness, learning environment, and academic and social self-perceptions [12–15]. Additional reported factors include the quality of student-instructor interactions, instructor performance, timely feedback, and institutional support, including for remote learning [15–17]. Individual-level characteristics, such as personality traits, gender, and ethnicity, have also been shown to affect student learning [18–20].

We used several graduate and medical education theories to interpret our findings. From the lens of curriculum review and reform, Biggs’ theory of constructive alignment provides a platform to examine how well aligned the intended program outcomes, learning activities, and assessment practices are with possible graduate career paths and thereby identify gaps that need to be filled. Our findings suggest that while the program aspires to prepare graduates for diverse professional pathways, much of the existing curriculum and structures have remained stagnant, still focused on traditional academic research careers. This represents a tremendous opportunity for growth and is echoed in our participants’ calls for expanded leadership, industry-engagement, and applied training opportunities that will feed their professional identity and provide transferable skills beyond their specific disciplinary expertise. In addition, systems thinking perspectives [21] allow us to appreciate the complexity of the institute and its programs and the challenges inherent in keeping pace with rapidly evolving scientific, healthcare, and labour-market environments. This framing highlights the importance of continuous program evaluation and iterative redesign and underscores the importance of themes and concepts that emerged beyond ‘course content’ throughout the program review exercise. Together, these theoretical perspectives provide a coherent structure for interpreting our findings and guiding future curriculum development.

Consistent with the findings of others above, our evaluation highlighted the critical role of program identity and structure, faculty engagement, and institutional support in contributing to student success and a positive learning experience. Beyond the expected concrete suggestions for changes to the program and its curriculum to meet the students’ needs as researchers, was the clear alignment of thoughts and ideas from all stakeholders with the new reality of life after graduate school. These findings are in keeping with research that shows that graduate students have a desire to engage with their learning more intently than undergraduate students. According to Lave and Wenger’s ‘situated learning’ theory [22], graduate students will place greater emphasis on the more social and interactive aspects of the learning experience to enhance their sense of belonging and professional identity.

This evaluation exercise and its findings reaffirmed the value of systematic, regularly scheduled review, offering insights, both strengths and challenges, that may otherwise have gone unrecognized. This experience reinforced our commitment to making structured, evidence-informed evaluations an integral part of our quality assurance practices. However, as emphasized in ‘Step 6: Ensure Use and Share Lessons Learned’ of the CDC Program Evaluation Framework, the true value of evaluation lies in translating findings into action. Findings alone have limited impact unless they inform clear changes in policy, curriculum, and practice. Accordingly, our institute has prioritized implementing corresponding action plans, monitoring their impact, and sharing lessons learned within and beyond the institution. The support from the leadership team at the institute facilitated the ongoing implementation of changes identified. Following the retreat held in 2022, many recommendations were implemented, and their impact evaluated, while work on other recommendations is ongoing and will be assessed during the next program evaluation cycle, scheduled for the 2026–2027 academic year as part of the institute’s quality assurance cycle. This recognition of the importance of routine and systematic program evaluation has been a critical contributor to the success of this initial program evaluation, motivating the establishment of processes and systems to support streamlined repetition of the process in the future. An analysis of ‘progress to date’ on outstanding items from previous rounds will be added to the process as well.

The CDC Program Evaluation Framework provided instrumental guidance in supporting a rigorous, robust, and meaningful evaluation process. Although originally developed for public health initiatives, its actions and evaluation standards aligned well with the institute’s values and offered the flexibility required for application in educational context [8–10]. This approach offers valuable insights for educators in similar contexts, demonstrating how the framework can be effectively adapted to guide program evaluation and curricular improvement in graduate biomedical and clinical research programs, adding to existing literature in the use of the CDC Program Evaluation Framework in education [23].

While the CDC model itself is a clear and user-friendly tool for underpinning educational program evaluation, we believe several factors augmented our success with the use of the tool and should be considered when implementing a program-wide evaluation similar to ours. These include: (a) buy in from the executive team of the institute; (b) the creation of a clear set of recommendations that were tied directly to the findings of the various studies within the review; (c) a structured retreat to convert recommendations into action plans; and (d) alignment between evaluation outputs and institutional quality assurance expectations. Further, the support from the institute’s leadership extended to the idea of embedding this kind of program evaluation into the established quality assurance cycle that was already in place. It was established that this program evaluation process would take place every six years in alignment with other externally mandated activities.

Although the steps in this model could be broadly applied for evaluation across other graduate programs in other post-secondary institutions, it is to be noted that some of the decisions made in regard to Steps 3 and 4 from this framework are specific to the context of our institute. For instance, as a result of the size of our program, we were not able to conduct multiple different focus groups on different topics, and instead chose to focus on CSPs.

As stated, one of the strengths of this study is its systematic approach to program evaluation, guided by the CDC Program Evaluation Framework. Another key strength is the high level of stakeholder engagement, particularly from students and faculty/supervisors. Their active participation enabled the study to go beyond describing the current state of the program by providing nuanced insights, identifying practical challenges, and generating actionable recommendations for improvement through robust quantitative and qualitative data, with similar challenges recognized across the stakeholder groups, strengthening the credibility of our findings. Overall, the study contributes to ongoing discussions in graduate education by highlighting the value of dynamic, responsive programs that align with the evolving needs of students.

A key challenge for graduate programs of this size and diversity is ensuring that the curriculum remains relevant and aligned with the goal of preparing future leaders in biomedical research. This is not a new challenge. Gutlerner [24] described similar challenges in a large US-based graduate biomedical program including the challenge of coping with the multiple, somewhat siloed, disciplines that students are drawn from. Although only about 26% of total students in the institute participated in the voluntary program evaluation, the sample included both doctoral and master’s students from diverse academic fields, and across all the research institutes, providing valuable insights into program strengths and areas for improvement. Given the program’s size and diversity, the relatively small but representative sample also yielded notably consistent findings across stakeholder groups, providing reassurance to the evaluation results. Of utmost importance is the rapidly evolving landscape of biomedical and clinical research. This implores the institute to remain responsive to the needs of students, supervisors, and faculty. Curriculum renewal efforts must be critically examined and, where necessary, dismantle these traditional paradigms that have historically shaped medical, biomedical, and translational science education.

This program evaluation has several limitations. First, participation in surveys, interviews, and the focus group was voluntary, which may introduce selection bias, as students who were more engaged with stronger opinions about the program may have been more likely to participate. Similarly, faculty members who are busy and less engaged may have been less likely to participate. While the survey was distributed through broad communication channels (e.g., newsletters, listserv, community platforms) with permitted forwarding intended to reach all relevant stakeholders, we cannot confirm that every individual saw the invitation, which may introduce unknown biases. However, we know that our institute consists of approximately 500 graduate students and 700 faculty members. Therefore, we estimate that around 26% (128/500) students and 12% (83/700) faculty members participated in the program evaluation. Between 2000 and 2020, our department had a total of 2,375 alumni. Second, only a single student focus group was conducted. Given the size and diversity of the student body, this represented only a subset of possible perspectives. However, this focus group was primarily intended to better understand student experiences with CSPs, which included around 26% of the students. This theme emerged from the student survey and was deemed notable to warrant further investigation. Nevertheless, additional focus groups could provide a more comprehensive understanding of student experiences across broader program areas. Finally, the program evaluation was cross-sectional capturing stakeholders’ experiences at a single time point. As a result, we cannot assess how the outcomes evolve over time as the recommendations are implemented. Further, the institute-specific recommendations, such as those related to the CSPs, may not be generalizable to a broader educational context. Nonetheless, this evaluation provided robust, data-driven insights, which guided actionable recommendations and will continue to inform future program refinements. Future studies could explore adapting our approach in broader educational contexts across institutions to assess generalizability and long-term outcomes.

## Conclusions

The mixed-methods evaluation identified consistent stakeholder priorities for improving the relevance and accessibility of training within a large biomedical graduate institute, including enhanced practical skills training (statistics, coding, writing), strengthened professional development and career supports that reflect the new career trajectories of our graduates, clearer program identity/structure, and strategies to improve faculty engagement. By presenting a transparent and traceable recommendation set and describing early utilization activities (implementation planning and establishment of an evaluation cycle), this work offers transferable processes that other institutions may adapt, while acknowledging context-specific constraints. Ongoing evaluation cycles will be essential to monitor the impact of implemented changes and sustain alignment between graduate education and evolving scientific and workforce needs.

## Supplementary Information

Supplementary Material 1.

## Data Availability

The program evaluation datasets used in this study are available from the corresponding author on reasonable request.

## Abbreviations

CSP: Collaborative Specialization Program
PhD: Doctor of Philosophy
MSc: Master of Science
REDCap: Research Electronic Data Capture
CDC: U.S. Centers for Disease Control and Prevention
