# Supplementary material for: Building a relevant biomedical graduate program: from review to reform
Source: BMC Med Educ. 2026 Apr 22;26:900. doi: 10.1186/s12909-026-09243-2 (PMC13235036; doi:10.1186/s12909-026-09243-2)

**Supplementary File 1:** Student survey questions


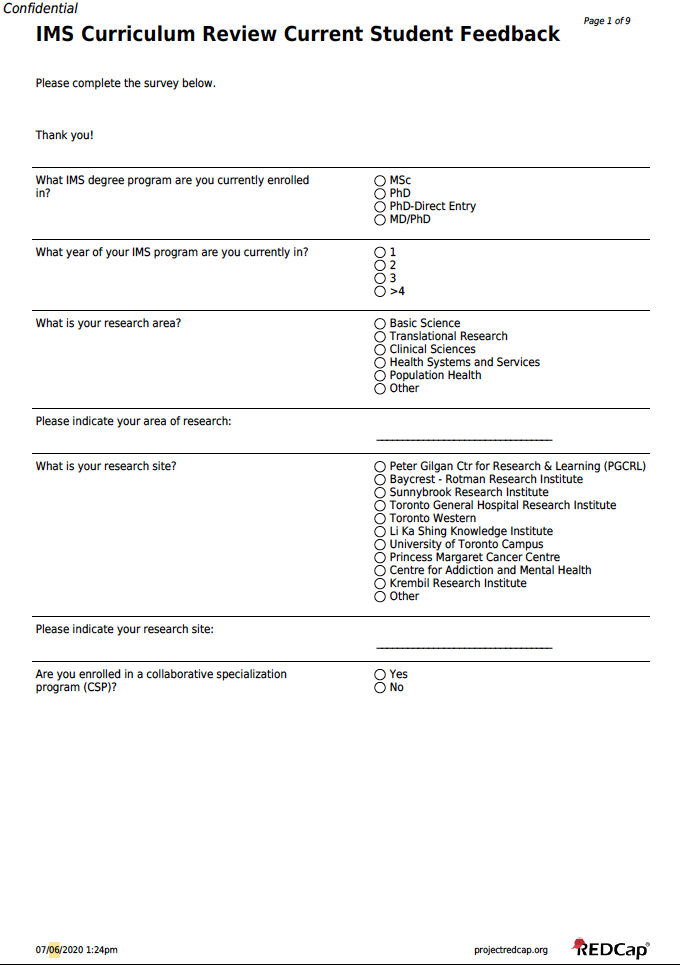


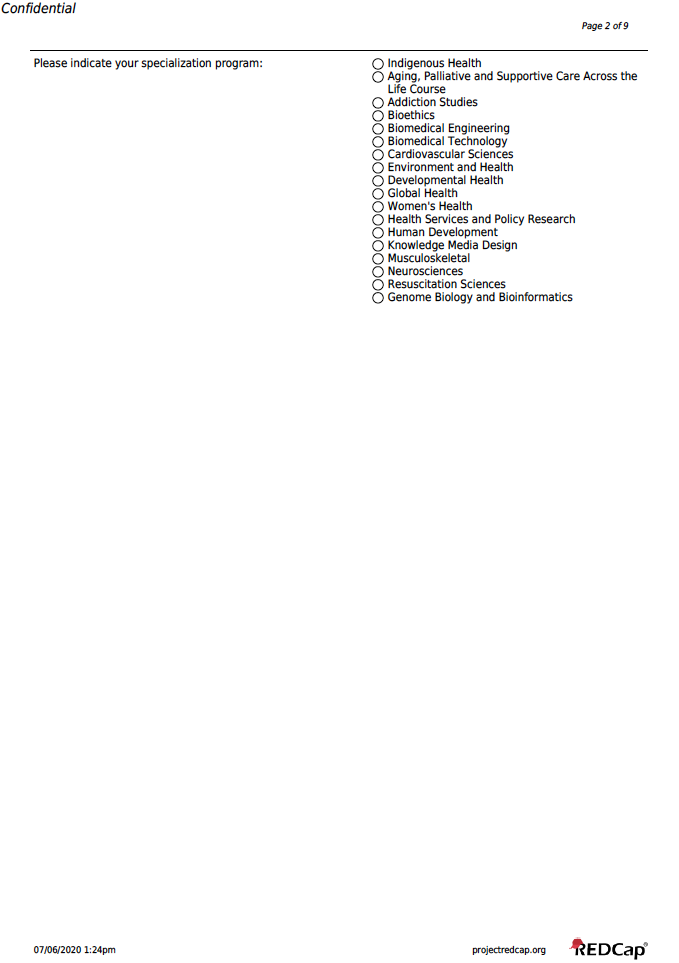


**
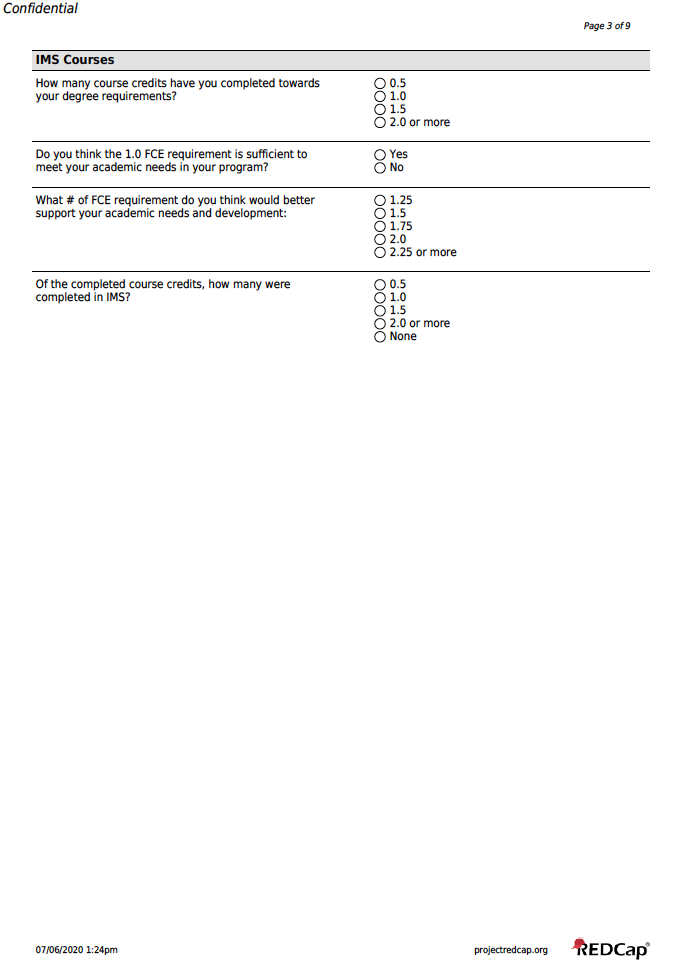
** **
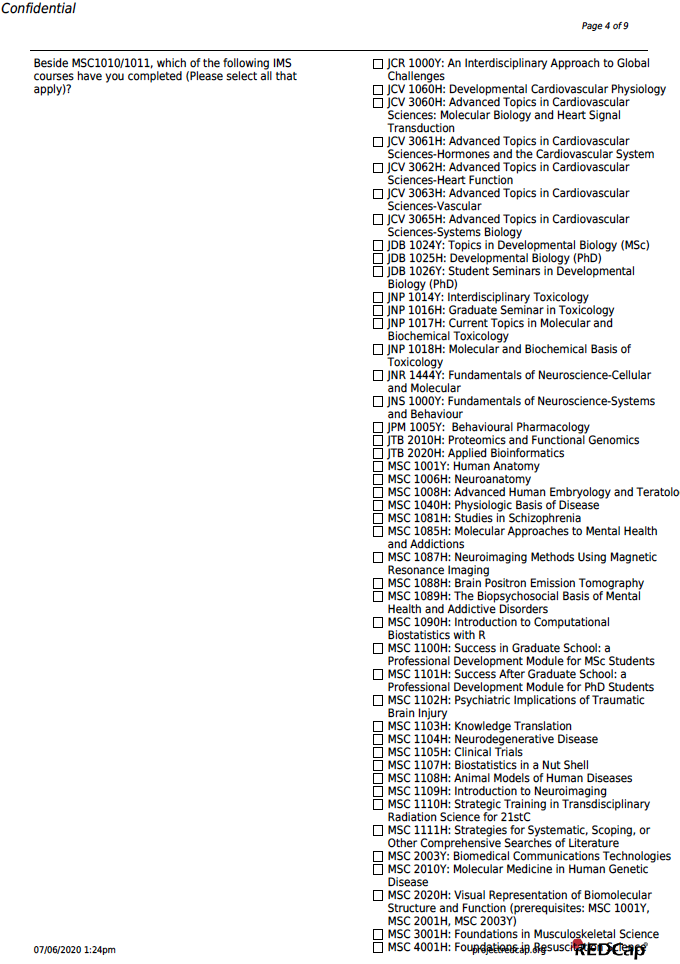
**

**
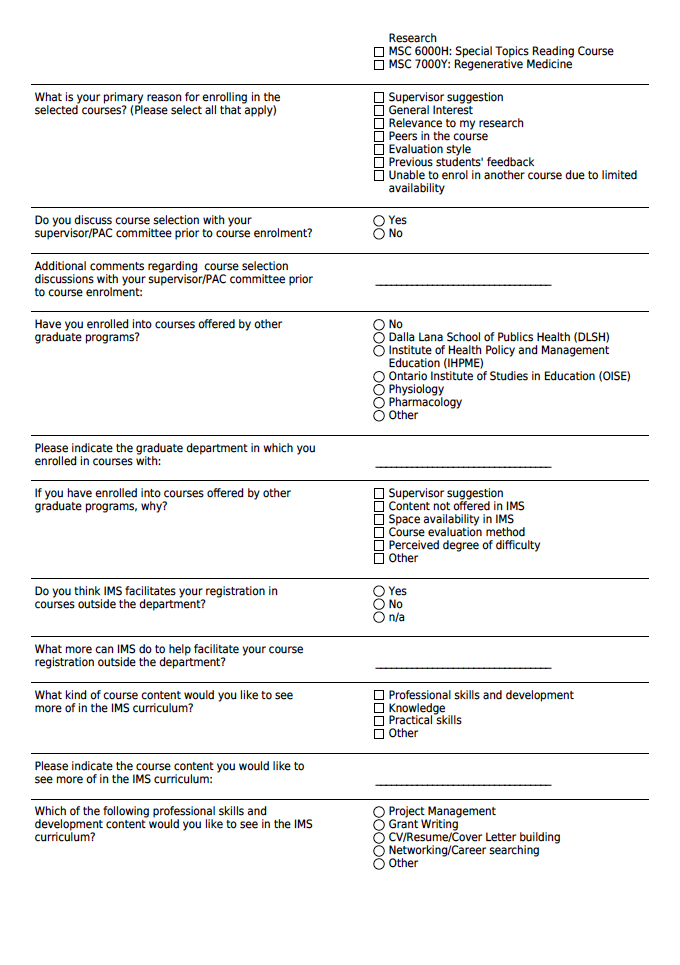
** **
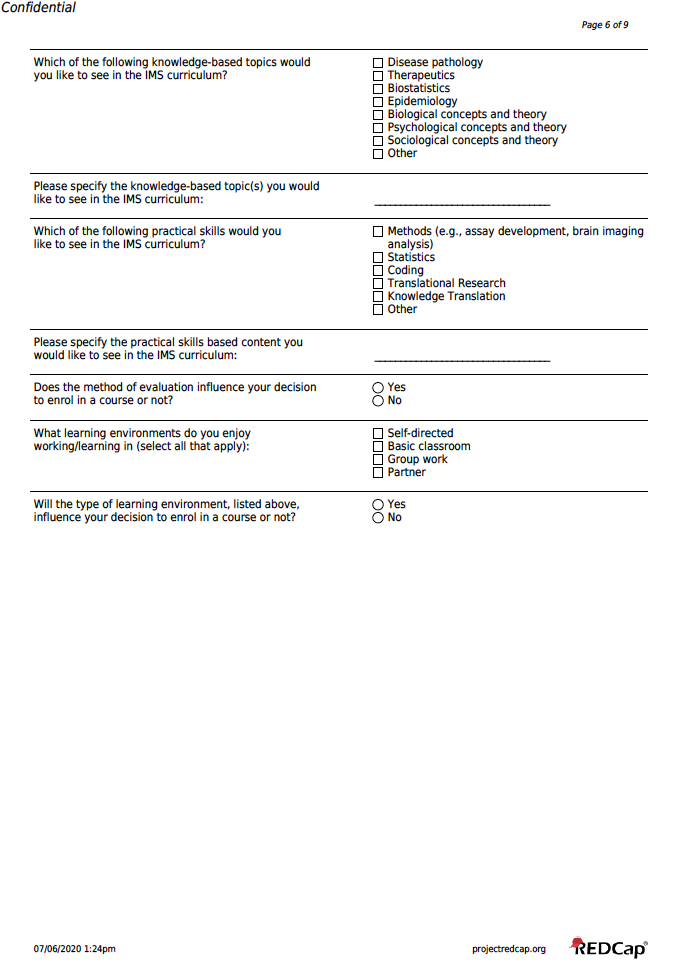
**
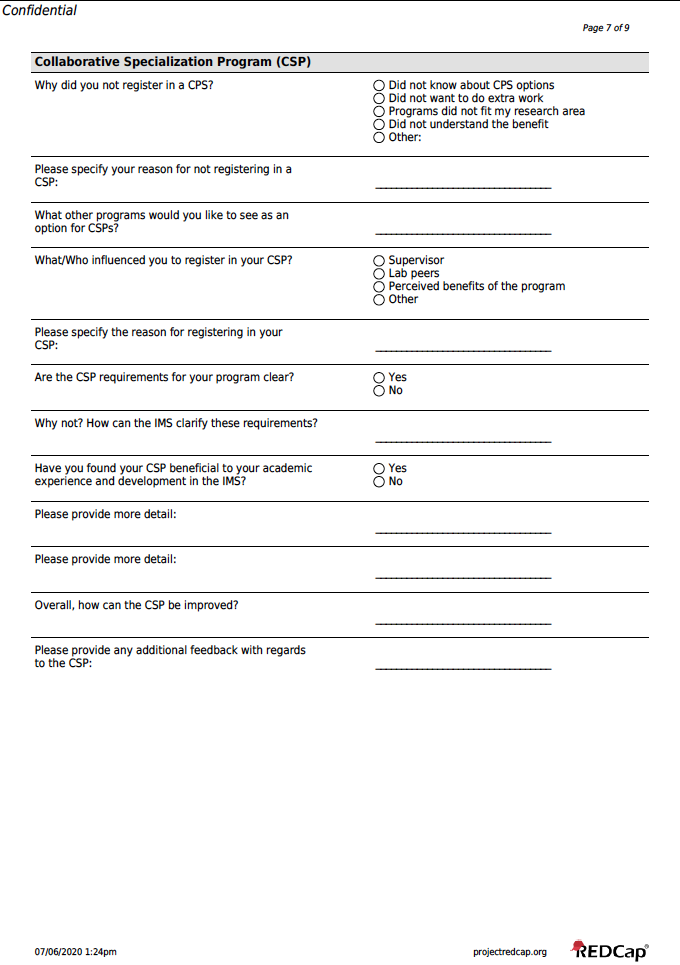

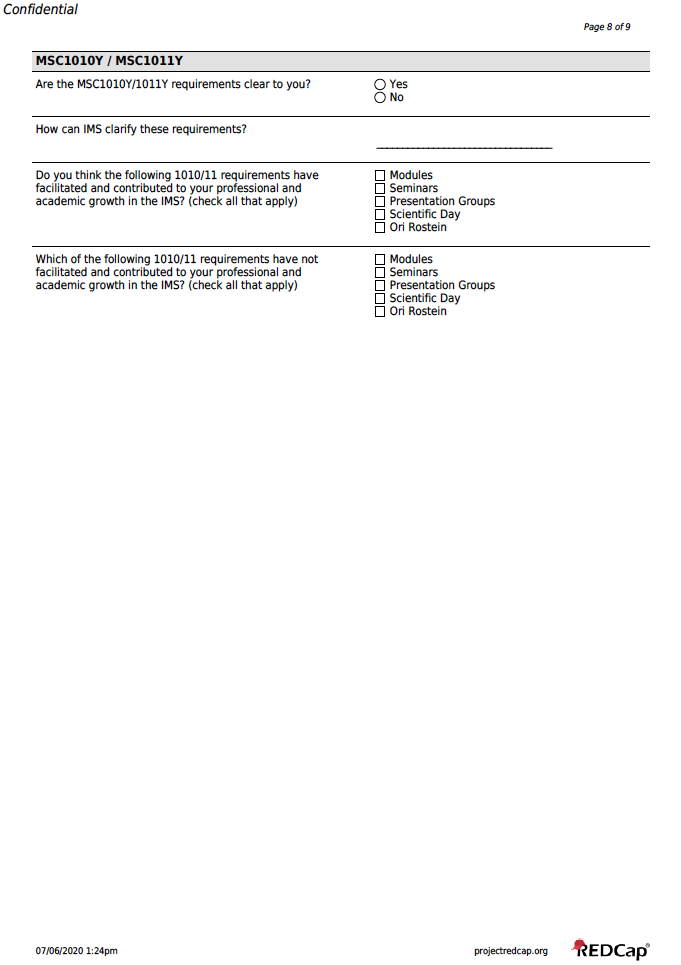

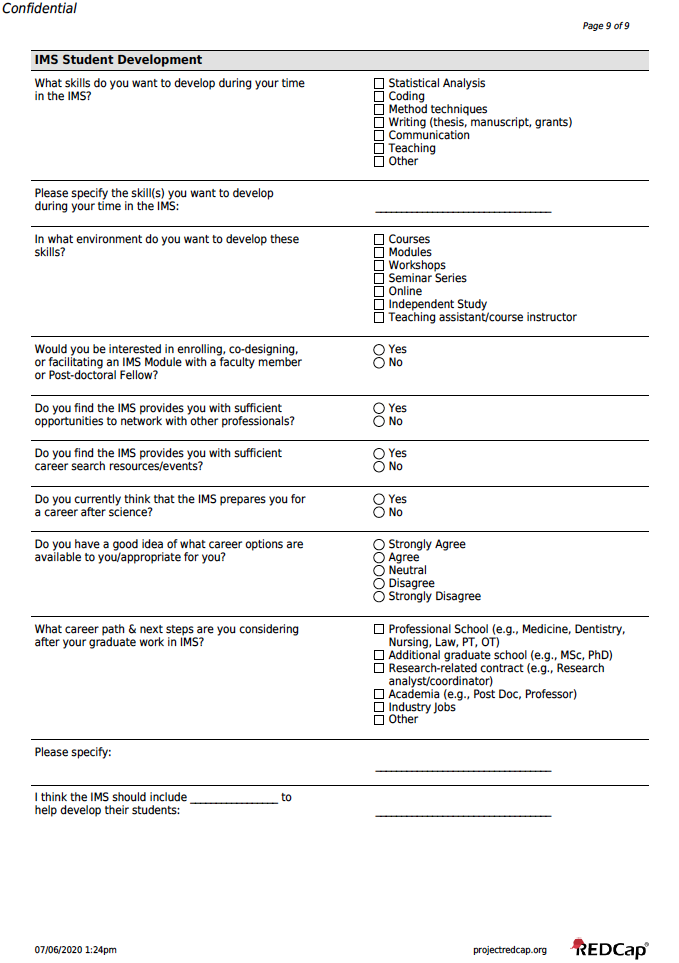


**Supplementary File 2:** Alumni survey questions


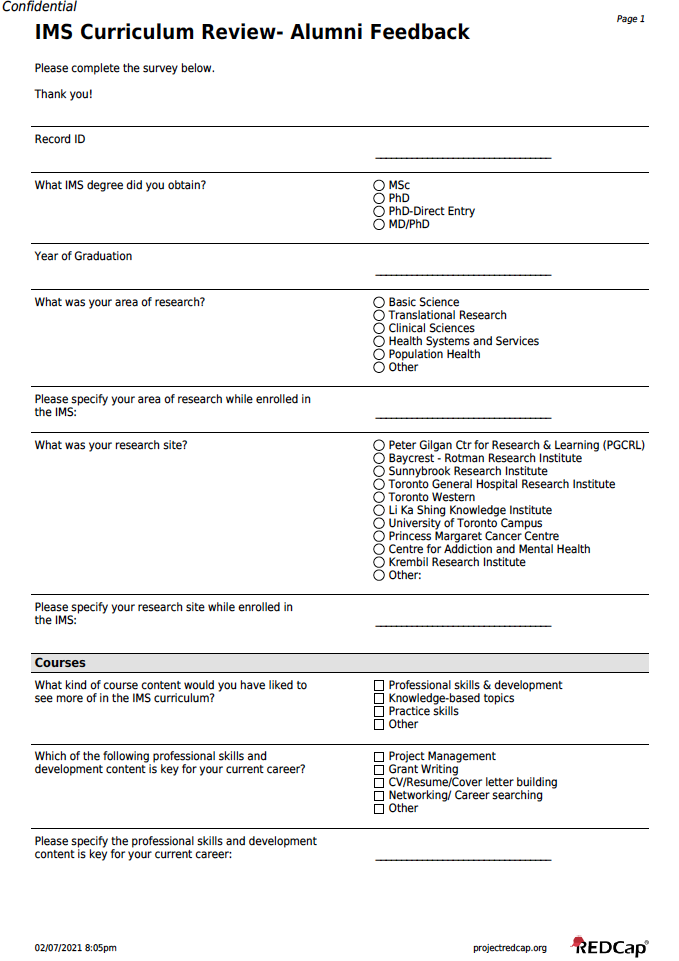


**
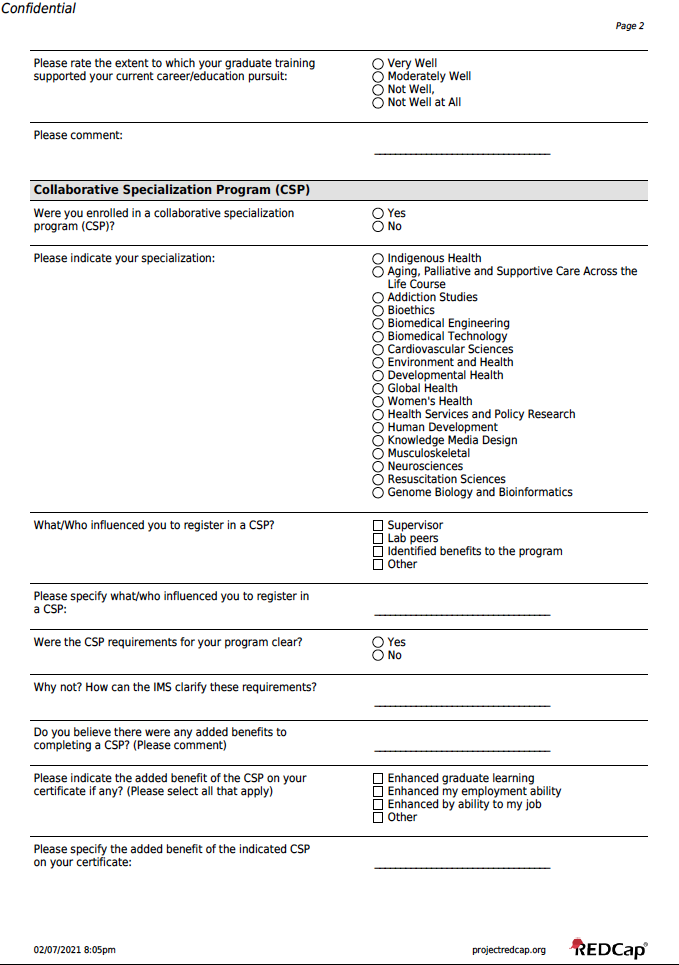
** **
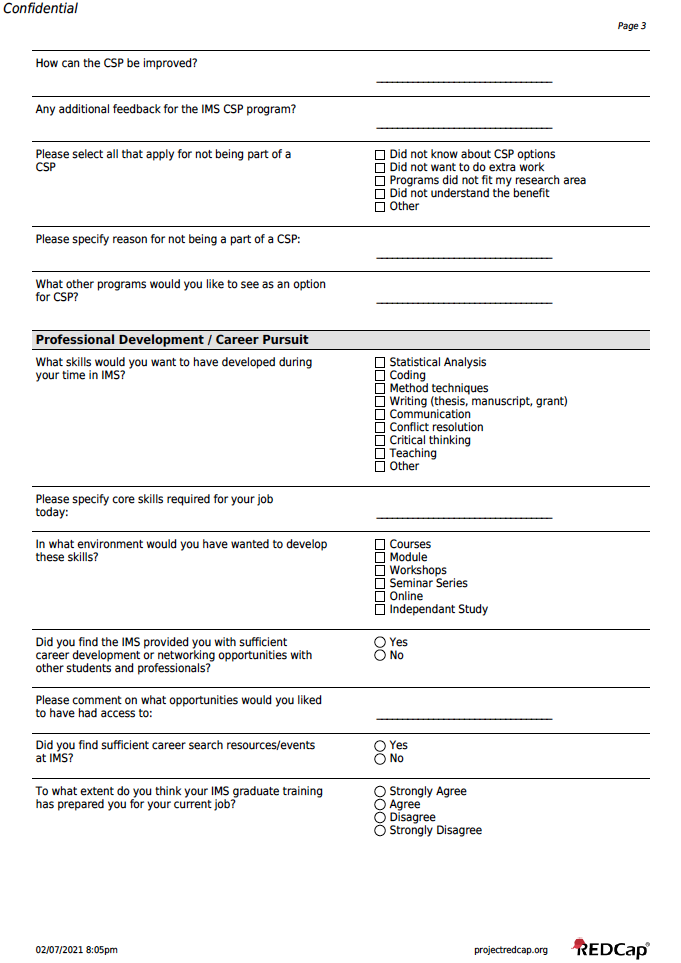
**
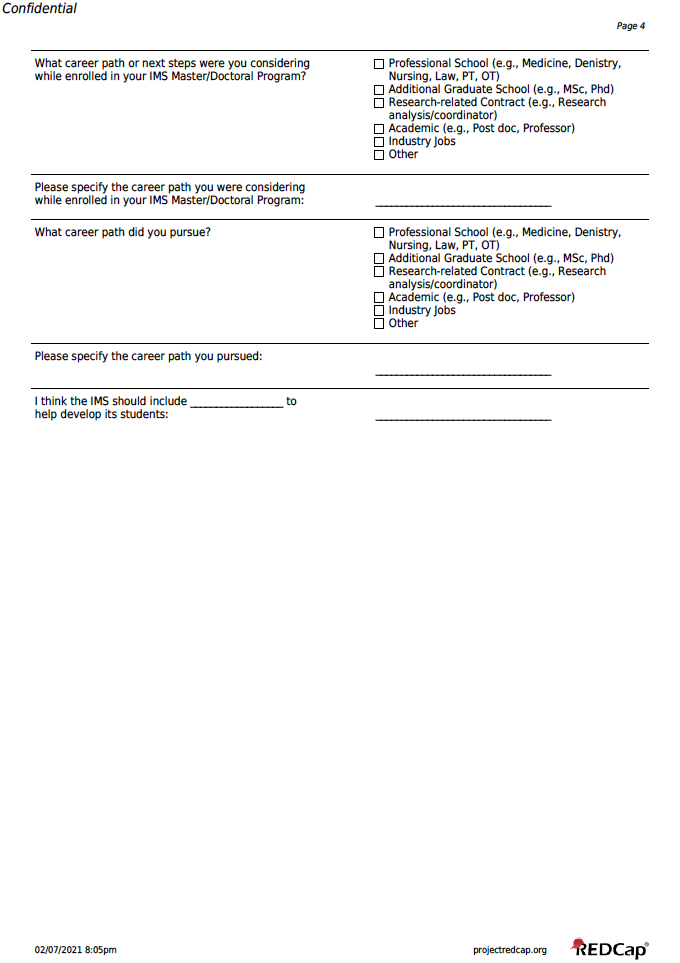


**Supplementary File 3:** Faculty/supervisor survey questions

**
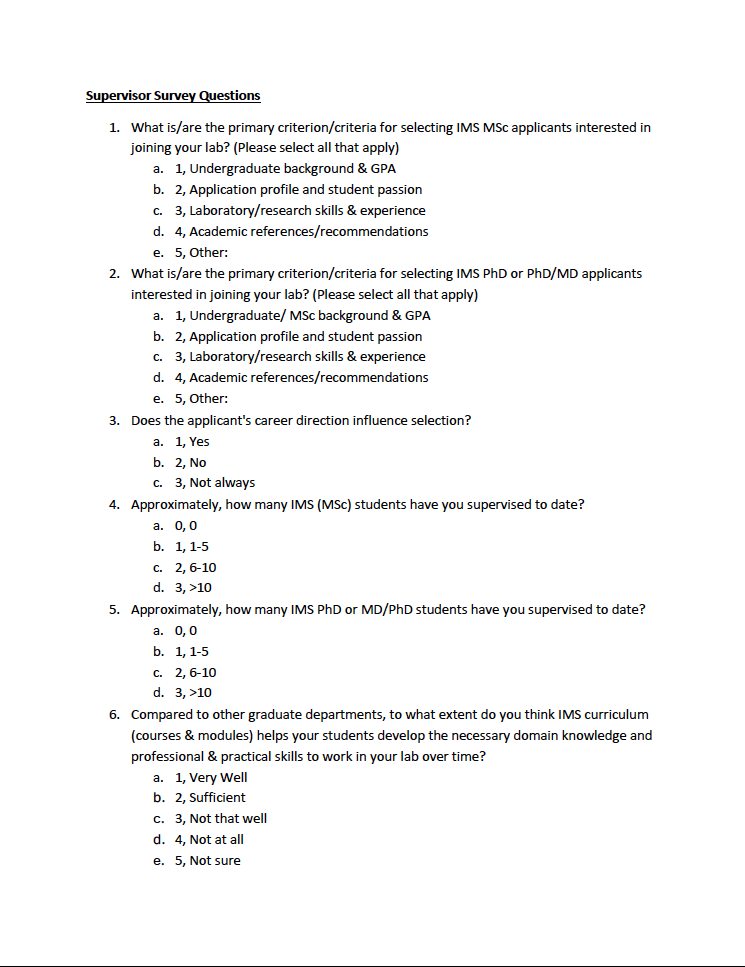
**

**
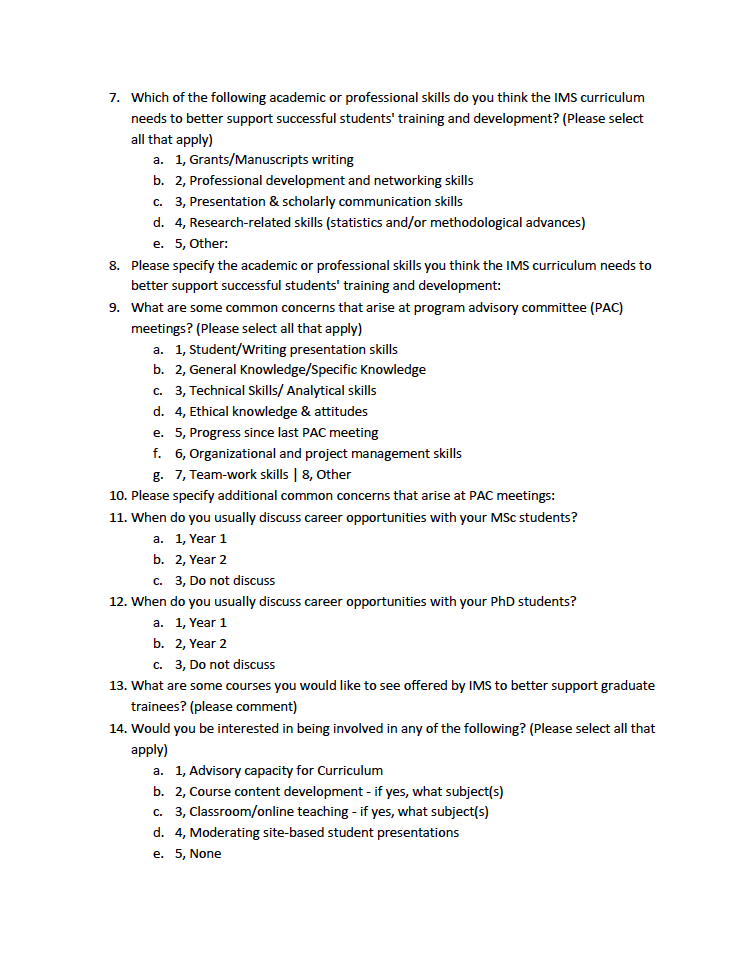
**
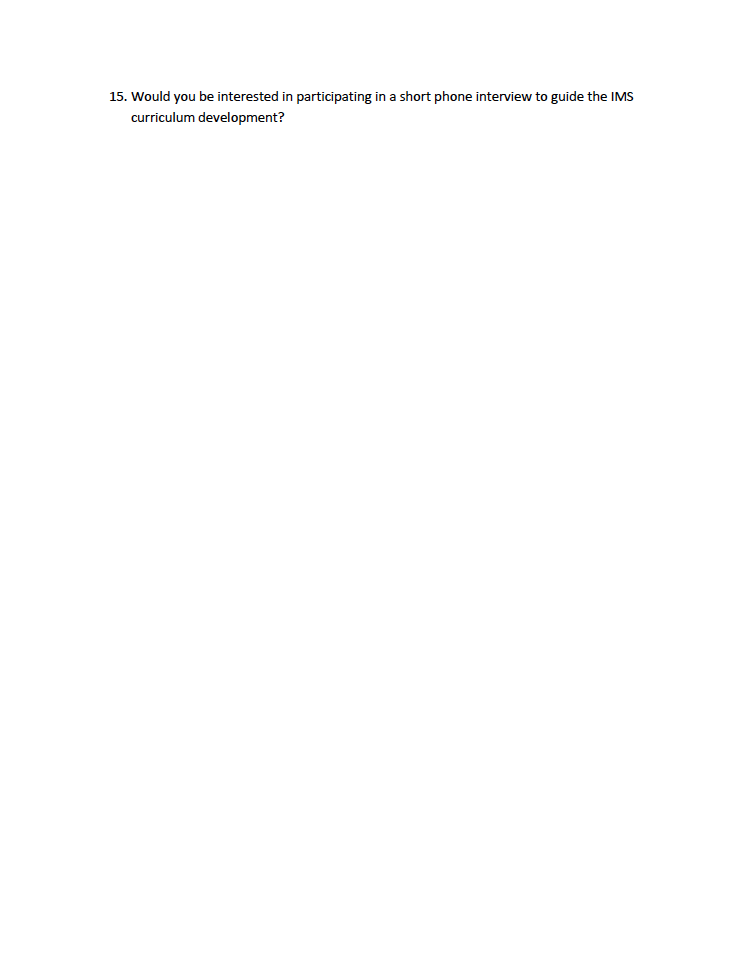


**Supplementary File 4:** Faculty/supervisor interview guide questions

1. Besides your experience as a supervisor, how have you been involved at the IMS (advisory, teaching, IMS committees)?
2. Would you be interested in being more engaged at the IMS? If so, how could the IMS enhance faculty engagement?
3. What are some curricular gaps (academic or professional) that the IMS needs to address? Why?
4. As a graduate program, what do you think the IMS program should focus on to be a distinguished program for students and faculty?
5. What do you think of IMS? What should it be? What do IMS students bring to the table that other students don’t?
6. What are some resources that IMS could establish to improve student and faculty experience?
7. Would you be willing to supervise students for independent courses?
8. Given the diversity of the content and research across the IMS, how can the IMS support students with unique learning needs?

**Supplementary File 5:** Student focus group questions


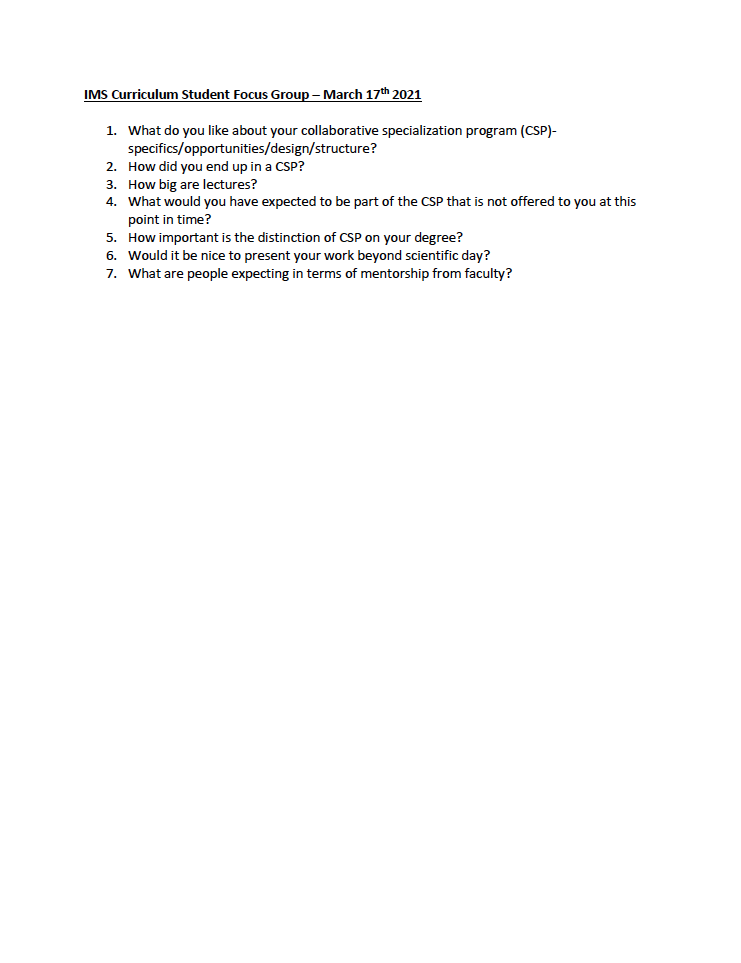

Supplement: Supplementary file 1 — Supplementary Material 1. [file 12909_2026_9243_MOESM1_ESM.docx]
